# It Is Not Necessary to Retrieve the Phonological Nodes of Context Objects for Chinese Speakers

**DOI:** 10.3389/fpsyg.2016.01161

**Published:** 2016-08-04

**Authors:** Qingfang Zhang, Xuebing Zhu

**Affiliations:** ^1^Department of Psychology, Renmin University of ChinaBeijing, China; ^2^Institute of Psychology, Chinese Academy of SciencesBeijing, China; ^3^Institute of Linguistic Studies, Shanghai International Studies UniversityShanghai, China

**Keywords:** speech production, phonological encoding, picture naming, word translation task, word association task

## Abstract

The issue of how activation is transmitted from semantic to phonological level in spoken production remains controversial. Recent evidences from alphabetic languages support a cascaded view. However, given the different architecture of phonological encoding in non-alphabetic languages, it is not clear whether this view applies in Chinese, as a non-alphabetic script. We therefore investigated whether the not-to-be named pictures activate their phonological properties in Chinese speech production. In Experiment 1, participants were presented a target English word and a context picture (semantically related or unrelated, phonologically related or unrelated to target word in Chinese) and were asked to translate the English word into a Chinese word. The translation latencies were faster in the semantically related condition than in the unrelated condition. By contrast, no difference between phonologically related and unrelated was observed. In Experiment 2, in order to promote participants phonological sensitivity in a word-translation task, we increased the proportion of phonologically related trials from 25 to 50%. In Experiment 3, we employed a word association task that was more sensitive to phonological activation of context objects than a word translation task. The phonological activation of context objects were absent again in Experiments 2 and 3. Bayes Factor analysis suggested that the absence of phonological activation of context pictures was reliable. Results consistently revealed that only target lemma could activate the corresponding phonological node to guide articulation whereas no phonological activation of non-target lemma’s in Chinese. The present findings thus support a discrete model in Chinese spoken word production, which was contrastive with the cascaded view in alphabetic languages production.

## Introduction

Speech production system is assumed to be as a network of interconnected nodes such as semantic, syntactic and phonological information (e.g., Dell, 1986; Roelofs, 1992; Caramazza, 1997; Levelt et al., 1999; Bloem and La Heij, 2003). The retrieval mechanism dealing with those units and structures is based on spreading activation (Collins and Loftus, 1975; Dell, 1986; Roelofs, 1992). In order to produce a target word, such as naming a picture, the activation in the semantic feature network spreads not only to the target but also to the semantically related concepts (semantic coordinates) via sharing the semantic features and the same category nodes. Subsequently, the co-activated concepts spread activation onto corresponding lexical nodes.

However, although most models postulate that any activated conceptual representation spreads activation to the lexical level, there are debates on how activation is transmitted from lexical nodes to phonological nodes. The *Serial discrete* models (e.g., Levelt et al., 1999) argue that for a given target word, only a single selected lemma spreads activation to the phonological level, and semantic processing must be completed before phonological processing commences. By contrast, non-serial models such as the *cascaded* models (e.g., Humphreys et al., 1988; Morsella and Miozzo, 2002) propose that multiple lexical-semantic candidates are co-activated during retrieval of the target word transmit activation to the phonological level. One of the debates concerns on whether the phonological activation is restricted to the target lexical node. To specify, when naming an object, does it necessarily lead to the phonological activation of multiple lexical nodes other than that of the target object only?

Empirical support for the serial models of spoken word production initially comes from an influential study reported by Schriefers et al. (1990). In a picture-word interference paradigm, participants are instructed to name a target picture while attempting to ignore a so-called “distractor” word superimposed on the target. A semantic relationship between a context word such as “dog” and a target picture such as “cat” slows naming relative to an unrelated word (e.g., “table”). Another typical finding is that a phonological relationship (such as the picture “cat” paired with the distractor “key”) speeds up naming responses (Glaser and Düngelhoff, 1984; Starreveld and La Heij, 1995). Most importantly, results show that semantic information is discretely prior to phonological information. Levelt et al. (1999) thus postulate that only the selected lemma could be phonologically activated. The follow-up investigation of mediated priming is used to account for the corresponding assumption via the use of “mediated” distractors, i.e., distractors which are phonologically related to a category coordinate of the target name. For example, for a target picture “dog”, a “mediated” distractor would be “can”, which is phonologically related to the target coordinate “cat”. An effect of such mediated distractors on latencies would imply that not only the target, but also co-activated items underwent phonological processing.

Recently, there is an increasing body of evidence for the cascaded models in alphabetic languages production such as English, German, Spanish, and Dutch (Morsella and Miozzo, 2002; Navarrete and Costa, 2005; Meyer and Damian, 2007; Kuipers and La Heij, 2009; Mädebach et al., 2011). The phonological activation of non-target objects is found in those studies. One of the most widely used paradigms is the picture-picture interference task. Speakers name a target picture while ignoring a superimposed context picture (target and context pictures have different colors so as to identify the target). Morsella and Miozzo (2002) manipulated the phonological relatedness between target and context pictures (i.e., the target is “bed”; a phonologically related context picture is “bell”; an unrelated picture is “hat”). Naming latencies were faster in the phonologically related condition than in the unrelated condition, reflecting that not only the phonological information of target picture, but also of the to-be-ignored context picture, are activated. The observation of phonological facilitation effect of context object has been replicated in English (Meyer and Damian, 2007), Spanish (Navarrete and Costa, 2005), and Dutch (Roelofs, 2008). This contradicts a central tenet of serial models of spoken word production and is in line with a cascaded view.

At the same time, cascading of activation from semantic to the phonological level seems is affected by different tasks. With a picture-picture interference task, Jescheniak et al. (2009) failed to obtain the phonological facilitation effect of context pictures during picture naming with Germany speakers. Jescheniak and Schriefers (1998) reported an mediated interference effect for pictures with near-synonymous names: when participants were instructed to name near-synonym target pictures with the dominant name, distractors which were phonologically related to the non-dominant name elicited interference effect (see also Peterson and Savoy, 1998, for related findings with regard to synonyms) However, Jescheniak et al. (2003) did not observe the mediated effects using a more sensitive electrophysiological measure. These findings indicate that cascaded processing is itself probably quite subtle.

One criticism is that different cognitive and attentional demands involve across tasks. For instance, in picture-picture interference task, two line drawings are superimposed in different colors, and speakers name a target picture based on color while attempting to ignore the context picture. It would be inevitable to pay attention the context picture because participants need to separate target from context picture (Bloem and La Heij, 2003; Navarrete and Costa, 2009). Roelofs (2008) proposed that the amount of cascading could be restricted by additional variables and different tasks. In a situation where context objects are visually similar to the target objects, more attention is allocated to the context objects compared with unrelated pairs, Oppermann et al. (2014) provide evidence for that the lexical and phonological representation of context objects could be activated only when they capture attention.

In a word translation task, native speakers were asked to translate a probe word from L2 to L1 while context pictures were manipulated phonologically related or unrelated to the response words. There was no phonological facilitation effect in Dutch-English and Spanish-Catalan speakers (Bloem and La Heij, 2003; Bloem et al., 2004; Navarrete and Costa, 2009), with an exception when the proportion of phonological related trials increased to certain level (50%) (Navarrete and Costa, 2009). In comparison with the picture-picture interference task, a word translation task can avoid the attentional problem. However, this task might be less sensitive enough to probe cascading phonological activation.

Humphreys et al. (2010) developed a word association task. In this task, a probe word and a context picture were presented and participants were asked to speak out the first word come to mind that was associated with the probe word as fast as possible, while ignoring the distractor picture. For example, in related condition, a probe word (“cobweb”) and a distractor picture (“spoon”) were presented, the to-be-produced target word “spider” was phonologically related to the context picture. In unrelated condition, a context picture was manipulated by sharing no phonological overlap with the to-be-produced target word (e.g., target word “spider” and distractor picture “hat”). Compared with unrelated condition, naming latencies were faster when the name of distractor picture was phonologically similar to the response target. Unlike the picture-picture interference task, a word and a context picture bear distinct physical attributes and could be separated easily. Thus, the cascaded transmission from semantic level to phonological level was not from the additional attention toward distractor pictures. The finding of 36 ms relatedness effect represented an effect size (Cohen’s d) of 0.31, which was comparable with the effect size (23 ms effect, *d* = 0.38, Meyer and Damian, 2007) in a picture–picture interference task. This indicates that this task is sensitive in exploring the cascaded semantic-to-phonological activation.

Overall, these findings indicate that not only the phonological information of the target, but also of the to-be-ignored context picture, is processed, which holds for the cascaded view assumes that any activated concepts and lexical nodes could transmit activation onto phonological nodes in alphabetic languages.

### Word Production in Alphabetic and Non-alphabetic Languages

Overall, the cascaded view has been supported by the evidences from alphabetic languages. However, relatively little attention has been paid to the possibility that whether the findings from alphabetic languages can be applied for non-alphabetic languages. Potential differences between languages concerning the architecture of phonological encoding might have consequences. O’Seaghdha et al. (2010) suggested that languages might differ in the “proximate unit” of phonological encoding (i.e., the primary selectable unit below the word level). In alphabetic languages, phonological segments constitute proximate units, and many phonological effects observed in different tasks are based on segmental overlap (e.g., Glaser and Düngelhoff, 1984; Schriefers et al., 1990). By contrast, the manipulation of phonological segments did not yield effects and instead priming effects were observed when syllables shared (e.g., Chen et al., 2002; O’Seaghdha et al., 2010; You et al., 2012). These findings in Mandarin Chinese and Cantonese suggest that syllables constitute the proximate units in non-alphabetic languages.

Potential difference across languages concerning the cascading phonological activation might related to the temporal courses of semantics and phonology in speech production. Electroencephalography (EEG) studies in spoken word production have shown that the temporal signatures associated with semantic and phonological processing are differ across languages (Abdel Rahman and Sommer, 2003; Dell’Acqua et al., 2010; Zhu et al., 2015). With an overt naming picture-word interference paradigm combined with EEG, Dell’Acqua et al. (2010) found significant differences on mean amplitude for both semantic and phonological relatedness in the time window of 250–450 ms. More importantly, the peak latencies of semantically related distractors (320 ms) coincided temporally with those of phonologically related distractors (321 ms). However, in Chinese, the semantic effect manifested itself in an early time window of 250–450 ms and was followed by a prominent phonological effect in a later time window of 450–600 ms, hence EEG results suggested a temporal dissociation between semantic and phonological stages in Chinese, reflecting that a serial model holds for information transmission in Chinese speech production (Zhu et al., 2015). The temporal courses of semantic processing were identical, whereas distinct temporal courses of phonological processing were observed across languages. These results led some support to the possibility that phonological encoding might differ in important aspects between alphabetic and non-alphabetic languages (i.e., Mandarin Chinese).

### The Present Study

In the present study, we investigated whether the cascaded view can be applied in Chinese, as well as potential differences between alphabetic and non-alphabetic languages. In Experiment 1, we employed a word translation task, in which an English word was accompanied by a picture that was either semantically related (or unrelated) or phonologically related (or unrelated) to the target Chinese word. The evidences for cascaded phonological activation in word translation task are restricted to alphabetic languages, such as English-Dutch translation (Bloem and La Heij, 2003) or Spanish-Catalan translation (Navarrete and Costa, 2009). We want to know whether this phenomenon can be replicated in a new population of Chinese-English bilinguals. This provides the opportunity to investigate whether there is cascaded activation of phonology from distractor pictures between alphabetic and non-alphabetic language contexts.

In Experiment 2, we aim to replicate the absence of phonological activation of context distractor pictures when the percentage of phonologically related trials increased from 25 into 50% during the entire session. The magnitude of priming effects could be larger when the proportion of related trials increased (de Groot, 1984; Navarrete and Costa, 2009). The high proportion of related trials would lead to a higher possibility to detect phonological priming (Navarrete and Costa, 2009). In Experiment 3, we employed a word association task, a more sensitive task, to tackle the cascading phonological activation.

According to the assumption of serial model, we predict a reliable semantic effect but the absence of phonological effect in Chinese spoken word production. According to the assumptions of cascaded model, we predict a semantic effect as well as a phonological effect.

### Ethical Statement

The current study was approved by the Independent Ethics Committee of the Institute of Psychology, Chinese Academy of Sciences in Beijing. Written consent was obtained from participants before the administration of the experiments.

## Experiment 1: Phonological Effect of Context Pictures Using Word Translation Task

### Methods

#### Participants

Twenty-two undergraduate students (9 Males; average 21.9 years; range 20–26 years) from Beijing Forest University and China Agricultural University were paid for their participation. They were native Chinese speakers of Chinese (L1) and highly proficiency in the English language (all started to learn English around 12 years old in the first year of middle school), and have normal or corrected-to-normal vision. We evaluated participants’ English proficiency on the basis of College English Test Band 6 (CET 6) (see also Liang and Chen, 2014; Zhang et al., 2014), which is an important examination in evaluating English proficiency in the public (Jin and Yang, 2006). All participants passed the test with the scores more than 550, representing a high proficiency English level.

#### Materials

In a word translation from English to Chinese task, eighty English probe words were selected from CELEX database. Their Chinese translation words as responses were generated correspondingly. Forty black and white line pictures were selected from a standardized picture database in Chinese (Zhang and Yang, 2003). All pictures had disyllabic names. Each picture was combined with two English words to form two different relationships between pictures and responses: semantically related and phonologically related.

To create semantically related relationship between pictures and responses, 40 pictures were paired with 40 English words. Picture names and responses words (Chinese) belonged to the same semantic category but had no orthographic or phonological overlap (i.e., “orange” as a probe, 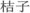 (/ju2zi5/) as a response in Chinese, 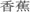 /xiang1jiao1/, “banana” as a context object). The same set of response words and pictures were then recombined to form semantically unrelated sets.

To create phonologically relationship between pictures and responses, 40 pictures were paired with other 40 English words. Picture names and response words (Chinese) shared syllable but differed in tone with the first character of the response words (i.e., “camera” as a probe, 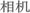 (/xiang4ji1/) as a response in Chinese, 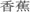 /xiang1jiao1/, “banana” as a context object). The same set of response words and pictures were then recombined to form phonologically unrelated condition. Therefore, A total of 160 English probe-Chinese response-picture stimulus sets were generated.

For English words as probes, average log frequency (taken from CELEX frequency in N-Watch, Davis, 2005) was 1.05 ± 0.57 per million for semantic condition and 1.62 ± 0.55 for phonological condition, phoneme length was 4.44 ± 1.61 for semantic condition and 4.89 ± 1.61 for phonological condition. For Chinese translation words as responses, average log frequency (taken from Chinese Linguistic Data Consortium, 2003) was 3.70 ± 0.55 for semantic condition and 4.40 ± 0.59 for phonological condition. All Chinese words were disyllabic.

#### Design

The experimental design included the distractor type (semantic vs. phonological) and the relatedness (related vs. unrelated) as within-participants and within-items variables. During the entire session, the order of items was pseudo-randomized for each participant with the constraint that a particular target did not re-occur for at least five trials, and the first phoneme of target words in the consecutive trials was not same.

#### Apparatus

The experiment was performed using E-Prime Professional Software. Pictures were standardized to a size of approximately 6 cm × 6 cm and displayed at the center of the screen. Probe words were presented in 28-point Times New Roman font, centrally superimposed on the target pictures. Naming latencies were measured from target onset using a digital voice-key, connected with the computer via a PST Serial Response Box.

#### Procedure

Participants were tested individually in front of a computer screen in a sound-proof room. Firstly, they were asked to familiarize themselves with the experimental stimuli by viewing the 80 English probe words with Chinese translation below. Then, participants were asked to translate those English probe words into Chinese words in isolation. Participants were corrected by an experimenter when they made an error. The incorrect trials were repeated at the end of series until participants produce correct response word. This is a typical procedure in word production study to ensure participants produce the correct response words (see also Bloem and La Heij, 2003).

After the familiarization phase, 160 experimental trials were presented. Each trial involved the following sequence: A fixation (+) presented in the middle of the screen for 500 ms, followed by a blank screen for 500 ms. Then, the probe word plus context picture was presented. Probe words and pictures disappeared when participants initiated a voice response. Participants were asked to translate the English probe word into Chinese as accurately and quickly as possible while ignoring the picture. An inter-trial interval was 2,000 ms. A short break was given after 80 trials. The experiment (including learning and testing phases) took about 40 min in total.

#### Results

Data from incorrect responses (2.33%), other responses such as mouth clicks (3.32%), naming latencies longer than 2,000 ms or shorter than 200 ms (2.95%), and those deviating by more than three standard deviations from a participant’s mean (2.36%) were removed from all analyses. The error rates were low and not analyzed further. **Table 1** displays the average naming latencies (Mean), standard deviations (SD) in ms and error rates (E%) by condition for Experiment 1.

**Table 1:** Average naming latencies (Mean), standard deviations (SD) in ms, and error rates (E%) by condition for Experiment 1.

Distractor type	Mean	*SD*	E%
Semantically related	1072	255	2.2
Semantically unrelated	1116	263	2.4
Phonologically related	1051	253	2.9
Phonologically unrelated	1044	254	1.8

The data were analyzed using a linear mixed effects model (Baayen et al., 2008) that included fixed effects of distractor type (semantic vs. phonological) and relatedness (related vs. unrelated), random effects for subjects and items. We used a maximal random effect structure a maximal random effect structure: for items, a slope for distractor type, relatedness, and their interaction; for subjects, a random intercept. Following Barr et al. (2013), we calculated the *p*-values for fixed effects by using model comparison with a simpler model with the same random effect structure but without the fixed effect in question. Results showed a significant main effect of distractor type (β = -27.4, *t* = -3.55, *p* < 0.001); the main effect of relatedness was not significant (β = 12.9, *t* = 1.54, *p* = 0.14); the interaction between distractor type and relatedness was not significant (β = 12.76, *t* = 1.26, *p* = 0.21).

For different distractor types, planned comparisons that assessed the effects of relatedness showed significant facilitation of semantic relation [β = -42.4, *t*(1575) = -1.99, *p* < 0.05, related minus unrelated, a slop for relatedness for subjects and items], while no effect of phonological relation [β = 8.7, *t*(1610) = 0.27, *p* = 0.80, related minus unrelated, a slop for relatedness for subjects and items].

A parallel analysis was conducted on the errors using a binomial family. Results showed no significant effects of semantic relatedness (*z* = 0.84, *p* = 0.40) and phonological relatedness (*z* = 0.10, *p* = 0.92).

The frequencies of English and Chinese words were different. To exclude the potential influence, we performed a linear mixed effects model (Baayen et al., 2008) including fixed effects of distractor type (semantic vs. phonological), relatedness (related vs. unrelated), and frequency (English vs. Chinese), and random effects for subjects and items. We used a maximal random effect structure: for subjects and items, slopes for all of the predictors (frequency, distractor type and relatedness) and the interactions of distractor type and relatedness. Results showed that there was no improvement in the fit when the full model with a three-way interaction of distractor type, relatedness, and frequency, compared with a model without the three-way interaction, *χ*^2^(1, 3185) = 0, *p* = 1 (for English word frequency) and *χ*^2^(1, 3185) = 2.02, *p* = 0.98 (for Chinese word frequency). Those results indicated that word frequency had no impact on latencies in the present study.

Before reaching the conclusion of no phonological effect, we need to evaluate the degree of confidence on the null hypothesis in these data. However, the traditional null hypothesis significance testing often implies that the null effect may due to lack of power rather than the truth of null effect. We thus conducted statistical tests using Bayesian analysis to quantify the strength of the evidence for the null compared to the alternative. Using the Jeffreys–Zellner–Siow (JZS) Bayes-factor paired *t*-test (Rouder et al., 2009), our result showed a Bayes factor of 4.10, suggesting the null hypothesis is 4.10 times more likely than the alternative hypothesis. According to the conventional interpretation of Bayes factor (3–10, “substantial”, Wetzels et al., 2011), the result showed substantial support for the null hypothesis rather than the alternative.

#### Discussion

The experimental results are consistent with previous studies using an identical task in alphabetic bilinguals (Bloem and La Heij, 2003; Navarrete and Costa, 2009). Compared with unrelated conditions, context pictures facilitated the translation of the probe words in semantically related condition while had no impact on the probe word translation in phonologically related condition, even using a totally different group of bilinguals in this experiment.

In line with previous studies on translation from the second language to the first language, the clear effect of semantic relatedness could be taken as evidence that the semantic representations of context pictures and probe words were activated (La Heij et al., 1996). Due to spreading activation semantically related context pictures facilitate the retrieval of the translation word at a semantic level. Those results ensure that it is valid to apply a word translation task to investigate processing underlying word production.

Nevertheless, the results showed that there was no phonological activation of context objects in Chinese speech production. On the one hand, when it comes to bilingual tasks, it is a necessity to ensure that those null effect wouldn’t be influenced by the level of L2 proficiency. According to the Revised Hierarchical Model (Kroll and Stewart, 1994), the connections between semantic and phonological nodes are weak in speakers with low L2 proficiency, and the connections become stronger in speakers with high L2 proficiency. The finding of semantic effect in our experiment confirmed that it is unlikely that the L2 proficiency was insufficient to influence the translation reaction time in different condition, especially excluding the impact on the absence of phonological effect.

On the other hand, the absence of phonological activation is inconsistent with the findings from cognate facilitation effect found in alphabetic bilingual groups (Costa et al., 2000) and phonological facilitation effect in picture naming tasks (Morsella and Miozzo, 2002). Although Bayesian analyses suggest the null hypothesis of no phonological effect being true, we need to evaluate the reliability of this result before reaching this conclusion. Especially, the empirical evidence on cascaded view has shown that the cascading activation may be weak.

## Experiment 2: Phonological Activation of Context Pictures when the Percentage of Phonologically Related Trials Increased

In Experiment 2, we aim to replicate the null effect of phonological relatedness under a more sensitive circumstance. Previous studies showed the percentage of related trials would influence the magnitude of priming effect (de Groot, 1984; Navarrete and Costa, 2009). Navarrete and Costa (2009) found phonological activation of context objects in high percentage of related trials (Experiment 1A) while no such activation in lower percentage of related trials (Experiment 1B). They interpreted it as the use of strategies during responses selection when the percentage of related condition is high. In other words, this manipulation would increase the possibility to obtain the phonological activation of context objects.

### Methods

#### Participants

Twenty-four undergraduate students (8 Males; average 23.3 years; range 21–26 years) from Beijing Forest University and China Agricultural University. They did not take part in Experiment 1.

#### Materials and Design

Identical to Experiment 1, with the exception that only phonological relatedness (related vs. unrelated) was included in this experiment. A total of 80 probe-response-picture sets were used.

#### Apparatus and Procedure

They were identical with Experiment 1, with the exception that all sets were presented twice in two separate sessions.

#### Results

Data from incorrect responses (2.0%), other responses such as mouth clicks (2.0%), naming latencies longer than 2,000 ms or shorter than 200 ms (3.7%), and those deviating by more than three standard deviations from a participant’s mean (1.02%) were removed from all analyses. **Table 2** displays the mean pictures naming latencies and standard errors for each by relatedness in Mandarin.

**Table 2:** Average naming latencies (Mean), standard deviations (SD) in ms, and error rates (E%) by condition for Experiments 2 and 3.

	Experiment 2			Experiment 3		
Distractor type	Mean	*SD*	E%	Mean	*SD*	E%
Phonologically related	901	207	1.8	793	174	8.2
Phonologically unrelated	902	218	2.2	790	160	9.4

Following Barr et al. (2013), we performed a linear mixed effects model that included fixed effects of phonological relation (related vs. unrelated), and random effects for subjects and items (a maximal random effect structure: for items, a slope for relatedness; for subjects, a random intercept). Result showed the effect of phonological relation was not significant [β = -1.94, *t*(3598) = -0.09, *p* ≤ 1]. Bayesian analysis as carried out in Experiment 1 resulted in a Bayes factor of 6.34, suggesting the null hypothesis is 6.34 times more than the alternative hypothesis. The result showed substantial support for the null hypothesis rather than the alternative.

A parallel analysis was conducted on the errors using a binomial family. Results showed no significant effects of phonological relatedness (*z* = -0.25, *p* = 0.80).

#### Discussion

Results showed no differences in naming latencies between conditions. Namely, the phonological overlap between context pictures and the to-be-produced responses had no impact on the translation processing. The absence of phonological effect in both Experiments 1 and 2 revealed that this finding is reliable. More importantly, it could hardly be attributed to lack of power based on the results from Bayesian analysis.

However, when it comes to the sensitivity of task, it should be noted that it is possible that the translation task itself may be less sensitive than naming task regarding the investigation of phonological activation of context objects. Especially, the phonological priming of context objects was obtained in picture-picture naming task (Morsella and Miozzo, 2002) while absent in translation tasks (Bloem and La Heij, 2003) in alphabetic languages. It should be noted that the finding of absence of phonological priming of context objects is in need of empirical evidence from more sensitive production tasks.

## Experiment 3: Phonological Activation of Context Pictures in a Word Association Task

### Methods

#### Participants

Twenty undergraduate students (2 Males; average 22.5 years; range 19–28 years) from Beijing Forest University and China Agricultural University. They did not take part in Experiments 1 or 2.

#### Materials

Twenty-five black and white line pictures were selected from a standardized picture database in Chinese (Zhang and Yang, 2003) as context pictures. Twenty-five probe words were selected. In the experiment, participants were asked to speak out the first word (response) that came to mind when they see the probe word. Generally, response words were semantically associated with probe words. The experiment manipulated the phonological relationship between picture and response word. Each picture was combined with one probe word to form the phonologically related pairs with response word.

In order to guarantee participants can produce the required response words, a pretest was carried out. First, we selected 68 potential response words and 68 pictures. These words shared syllables but differed in tone with the first character of 68 picture names (i.e.,
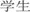
(/xue2sheng1/, student) as a target word, 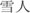
(/xue3ren2/, snowman) as a picture). Second, we selected probe words which were semantically associated with response words. Subsequently, 20 participants were presented with 68 probe words consecutively, and were asked to speak out the first word that came to their mind. We chose one response word if its production probability was higher than 80% among 20 participants. By this way, we selected 25 probe-response-picture sets as stimuli. The 25 probe-response-picture stimuli sets were used in the phonologically related condition. The same response words and pictures were then recombined to form phonologically unrelated conditions. These participants did not participated in the below experiment.

#### Design

The experimental design included the phonological relationship between response words and context pictures (related vs. unrelated) as within-participants and within-items variable. A total of 50 probe-response-picture trials were used.

#### Apparatus

The experiment was performed using E-Prime Professional Software. Probe word and picture presented simultaneously at the center of the screen. Probe words were presented in 28-point Song font. Pictures were standardized to a size of approximately 6 cm × 6 cm. As in the study of Humphreys et al. (2010), the bottom of each word had a visual angle of 3 above the horizontal midline of the screen; the top of each picture appeared at 2 above the midline of the screen. Naming latencies were measured from target onset using a digital voice-key, connected with the computer via a PST Serial Response Box.

#### Procedure

Each trial involved the following sequence: A fixation (+) presented in the middle of the screen for 500 ms, followed by a blank screen for 500 ms. Then, the probe word plus context picture was presented. Probe words and pictures disappeared when participants initiated a voice response. Participants were asked to speak out loud the first word that came to their mind as fast as possible while ignoring the context picture. An inter-trial interval of 2,000 ms concluded each trial. The experiment took about 15 min in total.

#### Results

Data from incorrect responses (8.8%), other responses such as mouth clicks (0.3%), naming latencies longer than 2,000 ms or shorter than 200 ms (0.35%), and those deviating by more than three standard deviations from a participant’s mean (1.65%) were removed from all analyses. **Table 2** displays the mean pictures naming latencies and standard errors for each by relatedness in Mandarin. Following Barr et al. (2013), we performed a linear mixed effects model that included fixed effects of relatedness (related vs. unrelated), by-subject and by-item random intercepts. Result showed the effect of phonological relation was not significant [β = -3.78, *t*(1778) = -0.59, *p* = 0.55].

Bayesian analysis as carried out in Experiment 1 resulted in a Bayes factor of 5.05, suggesting the null hypothesis is 5.05 times more likely than the alternative hypothesis. The result showed substantial support for the null hypothesis over the alternative.

A parallel analysis was conducted on the errors using a binomial family. Results showed no significant effects of relatedness (*z* = -1.11, *p* = 0.27).

#### Discussion

Results showed no differences in naming latencies between conditions, reflecting that the absence of cascading activation in Chinese. Interestingly, this word association task has been considered as a primary production task. The findings using this task in alphabetic language revealed more strongly cascading activation than typical picture–word interference tasks suggested (Humphreys et al., 2010).

## General Discussion

We performed three experiments to evaluate the phonological activation of distractor pictures. The results were as follows: (1) a phonological relationship between distractor pictures and target words has no impact on translation latencies; (2) the absence of phonological priming of distractor pictures is replicated when the percentage of phonologically related trials increases; (3) the phonological priming of distractor pictures is still absent in a more sensitive word association task. Our findings are novel in showing that speaking latencies were modulated by the semantic processing of distractor pictures only while no such effect regarding the phonological processing of context pictures in Chinese speech production.

Our results in Chinese showed the reliability of absence of phonological activation of context objects, regardless of task situations. Under normal manipulation (Experiment 1) and the enhancement of task sensitivity (Experiment 2, the phonologically related trials was increased), phonological overlap between target words and context objects didn’t affect the translation latencies. Most importantly, this pattern is replicated by a more sensitive word association task (Experiment 3) and a typical picture-picture interference paradigm (Qu, 2013). Results showed the manipulation of syllabic overlap between target and context picture names in Chinese had no impact on speaking latencies, indicating the phonological activation of target lemma only.

The results of Bayesian analysis revealed that the null effects could hardly to be attributed as a lack of experimental power. From the result obtained in Experiment 1, the clear effect of semantic effect confirmed the validity in speakers with high L2 proficiency (see Discussion in Experiment 1). Moreover, the semantic effect further excluded the possibility of materials in our experiments: the semantic effect indicated the activation of semantic representation of probe words and context pictures, there was no specific reason to assume that there is uncertainty about the probe words to undermine the results in phonological condition, especially when the identical context pictures were used in the entire experiment.

A possibility for the absence of cascading phonological activation might be from the variability of context pictures’ names. We thus calculated the *H* statistics of picture names used in experiments (Zhang and Yang, 2003). A picture that elicited the same name from every participant in the sample who was able to name it has an *H* value of zero, indicating that name agreements were perfect. A picture that elicited exactly two different names with equal times in the sample would have an *H* value of 1.00. The mean *H* statistic values are 0.56 in Experiments 1 and 2 (the same pictures were used in Experiment 2) and 0.75 in Experiment 3. The *H* values were relatively good in all experiments. In the present study, we did not ask participants to name pictures explicitly. Furthermore, we recruited two groups of participants to name pictures used in experiments. Results showed that the percentage of correct picture name responses was 98.41 ± 4.99% from 11 subjects (2 Males; average 23.3 years) for pictures in Experiment 1 and 96.89 ± 6.02% from 18 subjects (2 Males; average 22.1 years) for pictures in Experiment 3. These results indicate that our pictures have a good name agreement.

When taking a closer look at those findings in alphabetic languages and the current report, it is interesting to note that there are indeed several factors could modulate the phonological activation of non-targets. For instance, the amount of semantic activation of context objects (Oppermann et al., 2010), i.e., the mediated effect could be detected under the circumstance of near-synonyms in which multiple alternative lemmas are activated to almost identical degrees due to extreme semantic competition between the two alternatives (Jescheniak and Schriefers, 1998). A second possibility refers to the availability of processing resources, for instance, the phonological activation of context objects vanishes when the visual degradation of the context object or degradation of the target object decrease the available resources (Mädebach et al., 2011). However, for the current study, neither of those factors could have impact on the null effect of phonological activation.

We highlight the fact that the absence of cascading phonological activation in spoken Mandarin is in fact fully compatible with two sets of results that we recently reported. Zhu et al. (2016) factorially crossed semantic and phonological relatedness in a picture-word interference task with Mandarin speakers, and in contrast to numerous previous findings from speakers of Western languages, the two types of relatedness exerted a strictly additive relationship in Mandarin. Based on additive factors logic, this can be interpreted as indicating serial/discrete information transmission between semantic and phonological levels. Zhu et al. (2015) provided further evidence for a serial model via EEG and showed that with Mandarin speakers, semantic and phonological stages emerged in sequential corresponding time windows, which conflicts with comparable EEG studies conducted on speakers of Western languages where both stages appeared largely at the same time. Both sets of results are in line with the absence of cascading phonological activation, and point toward a serial transmission mode in Mandarin spoken word production.

The proximate unit of phonological encoding was syllables and segments in Chinese and alphabetic languages (O’Seaghdha et al., 2010; Roelofs, 2015). We therefore defined phonological overlap condition at the syllable level in the present study. There is no obvious reason why information transmission from semantic to proximate unit level should be cascaded in alphabetic languages such as English or Dutch, but serial in Chinese. Electrophysiological evidences suggest that temporal courses of phonological processing are differ between alphabetic and non-alphabetic languages (Abdel Rahman and Sommer, 2003; Dell’Acqua et al., 2010; Zhu et al., 2015). Hence, processing of phonological information could fundamentally differ between languages. Perhaps this is because in Western languages, relatively few segments combine to form a potentially unlimited number of lexical items, whereas in Mandarin, the number of syllables is much larger and so a discrete activation makes sense for syllables whereas the process is more continuous for segments.

What are the implications of the present findings for the current theoretical frameworks of word production? To produce a target word, a central issue concerns the activation time point when different word components become available to the speaker (Strijkers and Costa, 2016a). As postulated by serial models, the concepts and lemmas are activated regardless of a speaker’s intention, whereas the morphemes, segments of the target names are only activated when there is a goal-directed intention of a speaker (Roelofs, 2006). Thus it is obligatory to form the link between lemmas and forms toward goal-directed target in the speech production system. The semantic effect observed in Experiment 1 showed that the semantic information of context pictures was indeed activated during translation processing, regardless the intention of a speaker. The absence of phonological effect of context objects indicates that in Chinese speech production, only the link between lemma speakers want to verbalize and its corresponding phonological node is made. Together with the seriality of semantic and phonological activation in Chinese (semantic effect, 250–450 ms, followed by phonological effect, 450–600 ms, Zhu et al., 2015), those findings indicate that “a sequential fashion where each of the linguistic components involved in speaking has its specific time-course and dedicated processing center in the brain” (Indefrey and Levelt, 2004; Strijkers and Costa, 2016a, p. 484).

In the framework of serial models, to account for the increasing evidence on phonological activation of context objects and the near-simultaneous retrieval of lexical and phonological knowledge in the course of alphabetic language preparation (Pulvermuller et al., 2009) and production (Dell’Acqua et al., 2010), Strijkers and Costa (2016b) frame a new way to think about the dynamics of lexical access by extending and/or modifying the serial view: “modify the system by including parallel-distributed activation dynamics where the different components of a word become activated simultaneously” (Strijkers and Costa, 2016b, p. 526). Instead, there is need for more and better empirical tests to evaluate that the influence of task, context and attention on the processing of context objects. Importantly, to meet the urgent need of framing a general theory of word production, different language characteristic (e.g., the different phonological architecture across languages) must be taken into consideration.

We acknowledge that the assumption emphasis on the different phonological proximate units across languages is empirically insufficient to account for the divergence on information transmission across languages. Other phonological properties, such as phonological neighborhood density (Peramunage et al., 2011), the role of relatively low number of atonal syllables and unique properties of tones in Chinese (Roelofs, 2015), need to be access directly in the future. Ideally, our major contribution would be that the present results could provide new directions for thoughts and future research for the difference on the dynamics of activation flow across languages.

To summarize, we suggest that in Chinese spoken word production, only target lexical entries can under certain circumstances activate their phonological properties, hence provide support for a serial model in which information from semantic to phonological layer is discrete. This pattern was not in line with the cascaded view in alphabetic languages production. We speculated that the discrepancy on the dynamics of activation flow between alphabetic studies and the present one in Chinese may be relevant to differences on phonological architecture across languages. The underlying mechanisms are in need of more investigation from a cross-linguistic perspective.

## Author Contributions

QZ and XZ designed experiment and wrote the manuscript. XZ carried out the experiment and analyzed the data.

## Conflict of Interest Statement

The authors declare that the research was conducted in the absence of any commercial or financial relationships that could be construed as a potential conflict of interest.
